# Supplementary material for: Novel Driver Strength Index highlights important cancer genes in TCGA PanCanAtlas patients
Source: PeerJ. 2022 Aug 11;10:e13860. doi: 10.7717/peerj.13860 (PMC9375969; doi:10.7717/peerj.13860)
Supplement: Supplemental Information 2 [file peerj-10-13860-s002.zip › COHORTS/genes plots/cohorts/2021_8_16_14_9_distribution_cohorts_males_THCA.pdf]

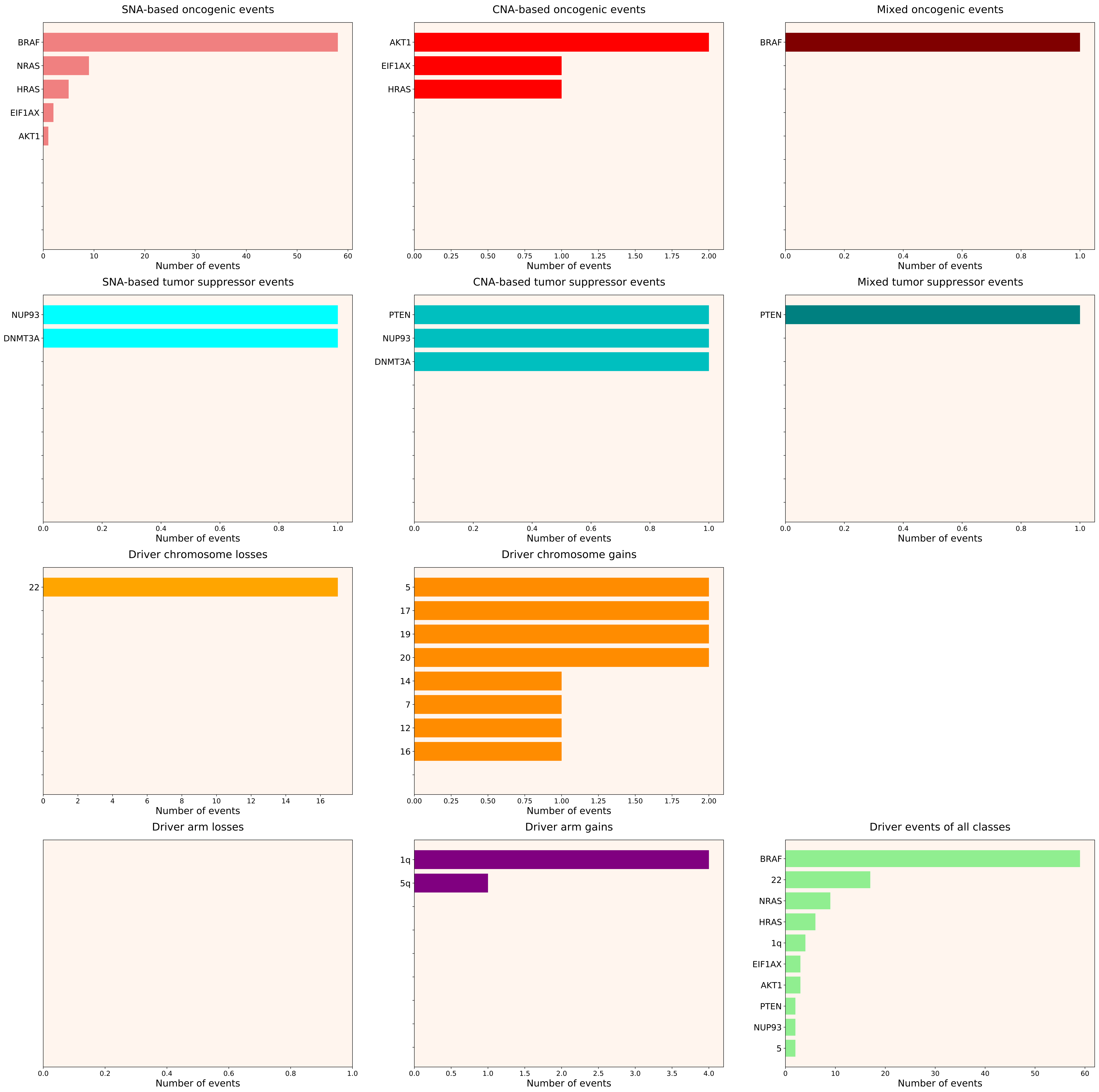

SNA-based oncogenic events
BRAF
NRAS
HRAS
EIF1AX
AKT1
0 10 20 30 40 50 60
Number of events
CNA-based oncogenic events
AKT1
EIF1AX
HRAS
0.00 0.25 0.50 0.75 1.00 1.25 1.50 1.75 2.00
Number of events
Mixed oncogenic events
BRAF
0.0 0.2 0.4 0.6 0.8 1.0
Number of events
SNA-based tumor suppressor events
NUP93
DNMT3A
0.0 0.2 0.4 0.6 0.8 1.0
Number of events
CNA-based tumor suppressor events
PTEN
NUP93
DNMT3A
0.0 0.2 0.4 0.6 0.8 1.0
Number of events
Mixed tumor suppressor events
PTEN
0.0 0.2 0.4 0.6 0.8 1.0
Number of events
Driver chromosome losses
22
0 2 4 6 8 10 12 14 16
Number of events
Driver chromosome gains
5
17
19
20
14
7
12
16
0.00 0.25 0.50 0.75 1.00 1.25 1.50 1.75 2.00
Number of events
Driver arm losses
0.0 0.2 0.4 0.6 0.8 1.0
Number of events
Driver arm gains
1q
5q
0.0 0.5 1.0 1.5 2.0 2.5 3.0 3.5 4.0
Number of events
Driver events of all classes
BRAF
22
NRAS
HRAS
1q
EIF1AX
AKT1
PTEN
NUP93
5
0 10 20 30 40 50 60
Number of events
